# Evolution of the Avian Eggshell Biomineralization Protein Toolkit – New Insights From Multi-Omics

**DOI:** 10.3389/fgene.2021.672433

**Published:** 2021-05-11

**Authors:** Nathalie Le Roy, Lilian Stapane, Joël Gautron, Maxwell T. Hincke

**Affiliations:** ^1^INRAE, Université de Tours, Nouzilly, France; ^2^Department of Innovation in Medical Education, University of Ottawa, Ottawa, ON, Canada; ^3^Department of Cellular and Molecular Medicine, University of Ottawa, Ottawa, ON, Canada

**Keywords:** birds, eggshell, calcium carbonate biomineralization, organic matrix, multi-omics tools, evolution

## Abstract

The avian eggshell is a remarkable biomineral, which is essential for avian reproduction; its properties permit embryonic development in the desiccating terrestrial environment, and moreover, are critically important to preserve unfertilized egg quality for human consumption. This calcium carbonate (CaCO_3_) bioceramic is made of 95% calcite and 3.5% organic matrix; it protects the egg contents against microbial penetration and mechanical damage, allows gaseous exchange, and provides calcium for development of the embryonic skeleton. In vertebrates, eggshell occurs in the Sauropsida and in a lesser extent in Mammalia taxa; avian eggshell calcification is one of the fastest known CaCO_3_ biomineralization processes, and results in a material with excellent mechanical properties. Thus, its study has triggered a strong interest from the researcher community. The investigation of eggshell biomineralization in birds over the past decades has led to detailed characterization of its protein and mineral constituents. Recently, our understanding of this process has been significantly improved using high-throughput technologies (i.e., proteomics, transcriptomics, genomics, and bioinformatics). Presently, more or less complete eggshell proteomes are available for nine birds, and therefore, key proteins that comprise the eggshell biomineralization toolkit are beginning to be identified. In this article, we review current knowledge on organic matrix components from calcified eggshell. We use these data to analyze the evolution of selected matrix proteins and underline their role in the biological toolkit required for eggshell calcification in avian species. Amongst the panel of eggshell-associated proteins, key functional domains are present such as calcium-binding, vesicle-binding and protein-binding. These technical advances, combined with progress in mineral ultrastructure analyses, have opened the way for new hypotheses of mineral nucleation and crystal growth in formation of the avian eggshell, including transfer of amorphous CaCO_3_ in vesicles from uterine cells to the eggshell mineralization site. The enrichment of multi-omics datasets for bird species is critical to understand the evolutionary context for development of CaCO_3_ biomineralization in metazoans, leading to the acquisition of the robust eggshell in birds (and formerly dinosaurs).

## Introduction

The avian eggshell is a calcitic biomineral that surrounds the telolecithal egg (i.e., possessing an uneven distribution of vitellus). The eggshell is essential to prevent desiccation during embryonic development and to regulate metabolic gas exchange. The shell is a remarkable physical barrier to protect the embryo against pathogens and mechanical shocks (Hincke et al., 2012; Gautron et al., 2021); moreover, the shell is a source of calcium for embryonic bone mineralization (Hincke et al., 2019). The egg is an autonomous source of all nutritive elements for embryo development, and therefore the unfertilized chicken egg is a high quality nutrient in the human diet. The study of the eggshell calcification process is of great importance to provide new insights into mechanisms of biomineralization, and to provide new tools to ensure the quality of the egg and its food safety for human consumption.

The oviduct is the organ of egg production in birds; it consists of six distinctly specialized segments that secrete the constituents of each egg compartment: infundibulum (vitelline membrane to enclose the egg yolk), magnum (secretion of egg white), white isthmus (elaboration of eggshell membranes), red isthmus (initiation of shell mineralization on mammillary cores), uterus (eggshell formation, cuticle deposition) and vagina (expulsion of mature egg). After ovulation of the ovocyte and its egg yolk, the egg white is secreted and deposited around the forming egg while it transits through the magnum segment. The eggshell membranes are deposited in the isthmus, and the calcitic eggshell is mineralized while it remains in the uterus (Nys et al., 2004). Eggshell mineralization takes place in an acellular uterine fluid secreted by uterine tissue, which contains mineral and organic precursors essential for shell mineralization (Gautron et al., 1997; Jonchère et al., 2012). *In vitro* crystallization tests using chicken uterine fluid have shown that this fluid strongly modifies the kinetics of calcite crystal formation and the resulting crystal morphology (Dominguez-Vera et al., 2000; Hernandez-Hernandez et al., 2008b). Finally, shell biomineralization is arrested with deposition of the phosphate-rich cuticle and the egg is laid. Eggshell mineralization follows five major stages: briefly, (1) amorphous calcium carbonate (ACC) is deposited on the entire surface of the outer eggshell membranes, which then (2) transforms into calcite at organic matrix clusters (mammillary cores); (3) calcite crystals nucleate from these sites and (4) grow rapidly with their *c*-axis becoming increasingly perpendicular to the eggshell surface. Two hours before egg expulsion, (5) mineralization is arrested and a thin layer of organic cuticle is deposited that covers the calcified layer and plugs the respiratory pores (Nys et al., 2004; Rodríguez-Navarro et al., 2015). For more detail regarding eggshell mineralization, the reader is referred to a new review by Gautron et al. (2021).

The avian eggshell is the result of an exceptional evolutionary strategy. Since the Late Devonian geological period (∼360 MYA – million years ago), the conquest of the terrestrial landscape challenged vertebrates to fulfill various vital functions such as breathing, locomotion and reproduction. Birds belong to the Sauropsida clade (that includes modern and extinct reptiles – turtles, lizards, snakes, crocodiles etc. – and birds) that appeared 315 MYA (Falcon-Lang et al., 2007). While amphibians have retained a need to lay their eggs in water, birds produce an impervious calcified barrier around the egg that they lay in terrestrial nests. Amongst laying-egg animals, birds possess the most solid eggshell; the soft-shelled eggs of turtles, lizards and snakes are less mineralized than bird eggshells, whereas crocodiles produce intermediate hard-shelled eggs (Choi et al., 2018). According to the fossil record, the first evidence of a calcified eggshell occurred at the Late Triassic/Early Jurassic, and belonged to a crocodilian (Bonaparte and Vince, 1979; Carpenter, 2000). In dinosaurs, the groups from which birds emerged, the oldest eggshells have been identified in the Early Jurassic, for which microstructural studies reveal a very thin shell (Garcia et al., 2006; Stein et al., 2019). According to numerous observations, the microstructure of dinosaur and bird eggshells is highly similar, with calcareous crystals forming an inner mammillary zone and outer palisade structure (Mikhailov, 1991). A recent study provided evidence for the independent evolution of calcified eggs in dinosaurs, with soft-shelled eggs as the ancestral character and the occurrence of at least three hard-shelled egg events (Norell et al., 2020).

**FIGURE 1:**
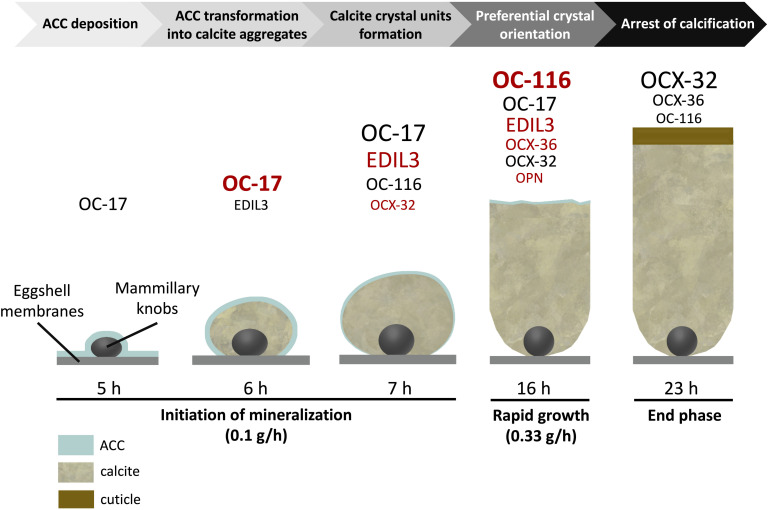
Schematic representation illustrating the presence of the 6 major proteins at the key events of chicken eggshell mineralization adapted from Marie et al. (2015a) and Gautron et al. (2021). Size of characters is relative to their level at individual stages [based on hierarchical ranking of the emPAI value from Marie et al. (2015a) for stages from 5 to 16 h and from Rose-Martel et al. (2012) – cuticle proteome – for the stage 23 h]. Overabundant proteins at each stage are indicated in red [based on spectral counting label free quantitative method and ANOVA statistical analysis from Marie et al. (2015a)].

Much knowledge of eggshell biomineralization has been obtained from studies utilizing the chicken egg. For decades, a combination of physical and biological approaches has led to increased understanding of this process. Microscopies (SEM and TEM), infrared and Raman spectroscopies, and X-ray diffraction have characterized the mineral phase (Rodríguez-Navarro et al., 2015; Pérez-Huerta and Dauphin, 2016; Choi et al., 2019). Protein purification, immunochemistry (Western blotting, colloidal gold immunocytochemistry) and proteomics were essential to identify occluded organic matrix constituents (Hincke et al., 2012; Gautron et al., 2021). Hundreds of proteins have been identified in eggshell proteomes from a small number of species (Table 1). Amongst this protein cortege, major functions have been assigned such as calcium-binding, matrix-organization, antimicrobial function, and so on (Marie et al., 2015a). The present review aims to describe six major proteins that have been identified as key actors in eggshell mineralization: Ovocalyxin-32 (OCX-32), Ovocalyxin-36 (OCX-36), Ovocleidin-116 (OC-116), Osteopontin (OPN), EGF (epidermal growth factor)-like repeats and discoidin domains 3 (EDIL3), and Ovocleidin-17 and its homologs (OC-17 and XCA). These six proteins possess essential functions (antimicrobial properties, regulation of CaCO_3_ crystallization or vesicular transport of ACC) and are present in significant abundance to be considered as major members of the eggshell biomineralization toolkit (Figure 1); it is likely that they were recruited during evolutionary acquisition of eggshell formation.

**TABLE 1:** List of eggshell proteomes and the presence of transcriptome/genome in bird and reptile species.

Species	Common name	Eggshell proteins	Uterine transcriptome/genome	Proteome references
**Neognathae → Galloanserae**				
*Gallus gallus*	Chicken	904	Yes/yes	Mann et al., 2006; Marie et al., 2015a
*Meleagris gallopavo*	Turkey	697	No/yes	Mann and Mann, 2013
*Numida meleagris*	Guinea fowl	154	Yes/no	Le Roy et al., 2019
*Coturnix japonica*	Japanese quail	622	Yes/yes	Mann and Mann, 2015
*Anas platyrhynchos*	Mallard duck	484	Yes/yes	Zhu et al., 2019
**Neognathae → Neoaves**				
*Taeniopygia guttata*	Zebra finch	475	No/yes	Mann, 2015
**Palaeognathae**				
*Struthio camelus*	Common ostrich	2	No/yes	Mann and Siedler, 2004
*Dromaius novaehollandiae*	Emu	2	No/yes	Mann and Siedler, 2006
*Rhea americana*	Greater rhea	2	No/yes	Mann and Siedler, 2006
**Crocodilia**				
*Crocodylus siamensis*	Siamese crocodile	58	No/no	Mikšík et al., 2018

*Hundreds of proteins have been identified in eggshell proteomes in a small number of species. The presence of uterine transcriptome characterization and/or genome annotation in these species was checked in NCBI databases.*

## Avian Eggshell: An Exceptional Vertebrate Calcium Carbonate Biomineral

**FIGURE 2:**
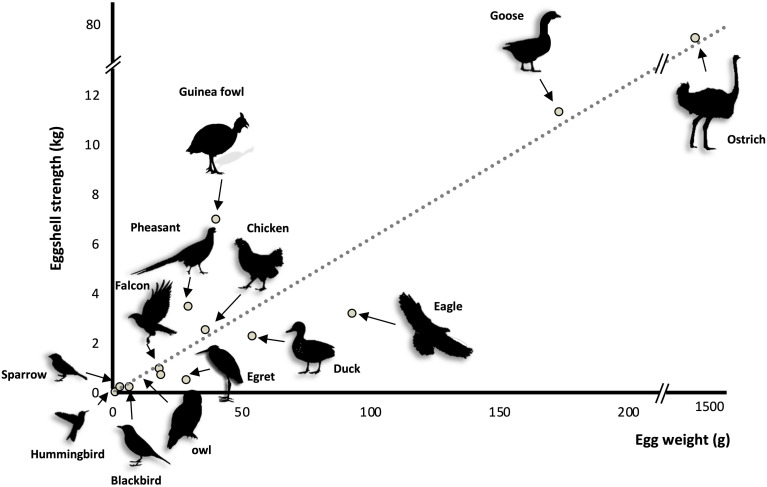
Eggshell strength as function of egg weight in thirteen bird species [adapted from Ar et al. (1979)].

The bird eggshell is a remarkable bioceramic that demonstrates exceptional mechanical properties; the eggshell appeared in the last common ancestor of amniotes around 326 MYA (Blair and Hedges, 2005; Ford and Benson, 2020). Across various bird species, eggshell strength is positively correlated with egg weight (Ar et al., 1979). Guinea fowl shows an elevated strength (Figure 2), which is related to its unique shell texture (Pérez-Huerta and Dauphin, 2016). In general, calcium carbonate crystal units of the bird eggshell palisade layer are parallel to each other and grow following the c-axis, i.e., perpendicular to the eggshell membranes and eventual eggshell surface (Hernandez-Hernandez et al., 2008a,b; Rodríguez-Navarro et al., 2015). This organization is also observed in fossilized dinosaur eggshells (Mikhailov, 1991; Voris et al., 2018; Dawson et al., 2020). In Guinea fowl (*Numida meleagris*), the first third of deposited shell is similar in structure to that of other species, whereas the outer two-thirds are composed of smaller crystal units with varying crystallographic orientations that form an intricate interlacing pattern that greatly improves shell strength (Petersen and Tyler, 1966; Panheleux et al., 1999a; Song et al., 2000; Pérez-Huerta and Dauphin, 2016). These animals are endemic to Central Africa and lay their eggs on the ground. The elevated breaking strength of their eggshell is likely the result of a specific adaptation to their environment (e.g., predation). A recent microstructural study of other bird eggshells suggested that Guinea fowl is not the only avian species to demonstrate this peculiar feature, since Rhea eggshell also has a similar crystalline organization (Choi et al., 2019). Since both are ground-nesting species, it is tempting to correlate their vulnerable nest location to the interlaced crystalline organization of their strengthened eggshell, which could better protect the egg. However, in other ground-nesting species, such as ostrich, chicken, turkey, etc., the eggshell ultrastructure is columnar.

The ultrastructure, polymorph and nucleation/growth of calcium carbonate crystals are controlled by a specific macromolecular toolkit, the organic matrix (OM) (Chien et al., 2008; Hernandez-Hernandez et al., 2008a,b; Zhao et al., 2013; Gautron et al., 2019, 2021). The bird eggshell OM represents 3.5% of the total shell weight including shell membranes. In the calcified part, the OM represents 1.5–2% of the overall contents (Panheleux et al., 2000). This eggshell OM is composed of proteins and proteoglycans, and has been studied for several decades, especially in chicken, *Gallus gallus* (Leach, 1982; Hincke et al., 1995, 2012; Pines et al., 1995; Gautron et al., 1997, 2021; Nys et al., 1999; Panheleux et al., 1999b; Arias and Fernandez, 2001). Eggshell membranes are the physical support for initiation of shell formation and are composed of a collagen-rich network of fibers (Wong et al., 1984; Arias et al., 1991; Ahmed et al., 2017). As in other metazoan biomineralization systems (e.g., molluscan shell, coral exoskeleton, echinoderm skeleton, etc.), the proteins of the OM possess various functional domains that regulate the matrix organization and control mineral formation (Marie et al., 2010; Ramos-Silva et al., 2013; Gautron et al., 2021). In addition, antimicrobial activities have been identified in avian eggshell that reinforce protection of the embryo against pathogens (Wellman-Labadie et al., 2008a,b,c; Cordeiro et al., 2013; Marie et al., 2015a, b; Gautron et al., 2021). Multiple studies of the evolution of calcium carbonate biomineralization in metazoans have demonstrated that certain homologous proteins were independently recruited to support this process, such as carbonic anhydrases and C-type lectins (Blank et al., 2003; Matsubara et al., 2008; Moya et al., 2008; Le Roy et al., 2014; Voigt et al., 2014; Karakostis et al., 2016; Weber et al., 2016). In other cases, newly arisen genes became specific and essential in the biomineralization process; for instance, the *Enam* gene product (Enamelin) in mammals is involved in dental enamel mineralization. This gene either emerged in mammals after the mammal/bird divergence or was lost in birds (Kawasaki et al., 2007). There are other examples of taxon-specific proteins involved in metazoan biomineralization, such as scleritin in the calcitic skeleton of octocorals (Conci et al., 2019; Le Roy et al., 2021), galaxin in the calcitic and aragonitic skeleton of corals (Conci et al., 2020), and pearlin in nacre of pearl oysters (Marie et al., 2011).

## Multi-Omics: A Significant Contribution to the Identification of Eggshell Organic Matrix Proteins

**FIGURE 3:**
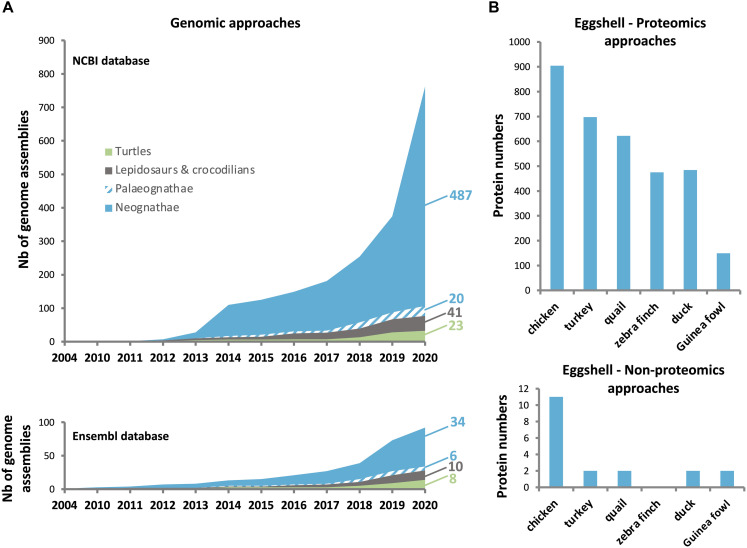
Number of genomes sequenced in birds and reptiles, and the proteins identified in eggshell of different avian species. **(A)** Increase in number of bird and reptile genomes available in NCBI (upper: https://www.ncbi.nlm.nih.gov/), and Ensembl (lower: http://www.ensembl.org/index.html) databases since the chicken genome assembly in 2004. **(B)** Number of identified proteins using proteomics (upper) and non-proteomics (lower) approaches. Data were compiled from Hincke et al. (1995, 1999), Panheleux et al. (1999b), Gautron et al. (2001, 2007),Mann et al. (2006), Mann and Mann (2013, 2015), Mann (2015), Marie et al. (2015a), Zhu et al. (2019), and Le Roy et al. (2019).

In the past few years, a huge enrichment of the genomic and transcriptomic databases in Aves has widely contributed to the identification of eggshell OM proteins in diverse species. The current Ensembl database^1^ contains genomes from 40 bird and 18 reptile species (Figure 3A), while NCBI lists genome assemblies from 507 bird (487 Neognathae and 20 Palaeognathae) and 64 reptile species. The 10,000 Genomes Project (B10K^2^) recently reported the genomes for 363 bird species including 267 new genomes, establishing a new pipeline to analyze the unprecedented scale of genomic data, and illustrating how these resources give improved resolution for genomic evolution analyses (Feng et al., 2020). The recent enrichment of genomic databases provides a critical tool for identification of bird eggshell proteome constituents by high-throughput mass spectrometry analysis (Figure 3B) (Mann et al., 2006, 2007a; Rose-Martel et al., 2012, 2015; Mann and Mann, 2013, 2015; Mann, 2015; Marie et al., 2015a; Gautron, 2019; Gautron et al., 2019, 2021; Le Roy et al., 2019; Zhu et al., 2019). Accurate gene annotation is critical to support proteomic approaches. For example, more than 1,300 chicken eggshell protein sequences with different identifiers were aligned to eliminate all redundancies; with this approach, 904 unique proteins were identified in the eggshell layers including membranes and cuticle (Gautron, 2019; Gautron et al., 2019). Another integrated analysis of chicken eggshell matrix enumerated a total of 676 eggshell matrix proteins in the mineralized shell (Yang et al., 2020). Additional bird eggshell proteomes have been studied quite extensively, identifying 697, 622, 475, and 484 proteins in the mineralized eggshell of turkey (*Meleagris gallopavo*), quail (*Coturnix japonica*), zebra finch (*Taeniopygia guttata*) and mallard (*Anas platyrhynchos*) eggshells, respectively (Mann and Mann, 2013, 2015; Mann, 2015; Zhu et al., 2019), and 149 proteins in the entire eggshell of Guinea fowl (*Numida meleagris*) (Le Roy et al., 2019). This low number of identified proteins in Guinea fowl eggshell is possibly due to incomplete annotation of its genome (NumMel1.0.).

The next section will describe the evolutionary context for six major proteins that have been identified as key actors in chicken eggshell mineralization (OCX-36, OCX-32, OC-116, OPN, EDIL3, and OC-17/XCA).

## Vertebrate Proteins Recruited in the Eggshell Biomineralization Process

### Ovocalyxin-32: An Antimicrobial Protein That Influences Eggshell Quality

Ovocalyxin-32 (OCX-32) was originally identified in chicken as an eggshell-specific protein, and its gene is highly expressed in the uterus and the isthmus regions of the oviduct (Gautron et al., 2001). This last study localized OCX-32 in the outer shell (outer palisade layer, the vertical crystal layer and the cuticle). Proteomic analyses revealed abundant OCX-32 in the uterine fluid during the initial phase of mineralization, and its relative enrichment in the palisade region of the eggshell (Marie et al., 2015b, a). OCX-32 was also identified in the proteome of the insoluble fraction of the chicken eggshell organic matrix (Mikšík et al., 2007). OCX-32 possesses 32% identity with mammalian carboxypeptidase inhibitors, latexin and the retinoic acid receptor-responder 1 (RARRES1). Recombinant OCX-32 inhibited bovine carboxypeptidase and the growth of *Bacillus subtilis* (Xing et al., 2007), suggesting an antimicrobial role for OCX-32 in providing protection to the developing avian embryo. Proteomic analysis of the chicken eggshell cuticle demonstrated that OCX-32 is one of the most abundant constituents of this non-mineralized region, and could play a major role in the antimicrobial properties of the cuticle (Rose-Martel et al., 2012; Bain et al., 2013). Polymorphisms in the gene coding for OCX-32, *RARRES1* (gene synonym*: OCX32*), are significantly associated with egg production traits (Uemoto et al., 2009; Romé and Le Roy, 2016). In another study, the quantitative trait loci (QTLs) on chromosome 9 were investigated in an F2 generation that was an intercross between two chicken lines divergently selected for eggshell strength (Takahashi et al., 2010). *RARRES1/OCX32* was identified as a candidate gene influencing eggshell quality (e.g., egg weight, egg dimensions and eggshell weight), and *RARRES1*/*OCX32* SNPs (single-nucleotide polymorphisms) are associated with eggshell quality and mammillary knob layer thickness (Dunn et al., 2008). Trait association studies of non-synonymous SNPs also revealed a significant effect of OCX-32 on shell color in white egg lines and line-specific significant effects on albumen height, early egg weight, puncture score, and yolk weight (Fulton et al., 2012).

**FIGURE 4:**
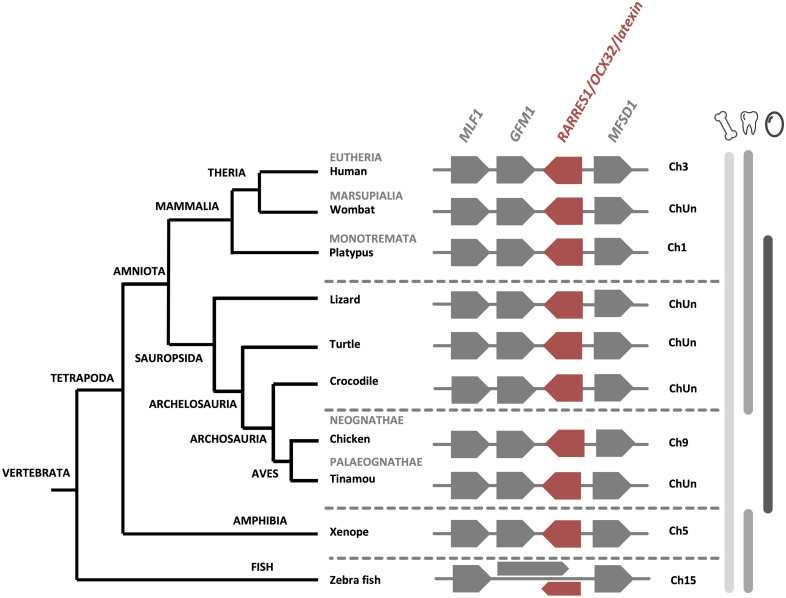
Synteny of *RARRES1/OCX32* genes in vertebrates. The *RARRES1/OCX32* gene is represented by a red box and the flanking genes *MLF1* (myeloid leukemia factor 1), *GFM1* (G elongation factor mitochondrial 1) and *MFSD1* (major facilitator superfamily domain containing 1) are represented by gray boxes. ChUn, chromosome unknown. Gene IDs are listed in Supplementary Table 2. The left part of the figure depicts the phylogenetic relationship between vertebrate species (adapted from www.tolweb.org and Kapusta et al., 2017).

Recent proteomics analyses failed to identify OCX-32 in the turkey and quail eggshell (Mann and Mann, 2013, 2015). Nevertheless, in addition to the chicken eggshell organic matrix, this protein has been identified in the eggshell proteome of zebra finch, Guinea fowl and mallard duck (Mann, 2015; Le Roy et al., 2019; Zhu et al., 2019). Synteny analysis of the *RARRES1/OCX32* gene using NCBI database, demonstrates that it is homologous from fishes to mammals (Figure 4), suggesting a common ancestor in vertebrates. In addition, the chromosomal location of the *RARRES1/OCX32* gene is highly conserved in a syntenous gene locus from fishes to mammals (Figure 4). Therefore, while OCX-32 is highly conserved and may be an important member of the eggshell mineralization toolkit, it does not appear to be present in all eggshell proteomes, nor is it unique to calcium carbonate biomineralizing organisms.

### The LBP/BPI/PLUNC Family Protein, Ovocalyxin-36

Ovocalyxin-36 (OCX-36) is a protein belonging to the bactericidal/permeability-increasing (BPI), lipopolysaccharide-binding proteins (LBP), and palate, lung and nasal epithelial clone (PLUNC) protein family (Chiang et al., 2011; Krasity et al., 2011; Baron et al., 2013). OCX-36 was first identified in the chicken eggshell; expression of its gene was detected in the uterus and to a lesser degree in red isthmus, which are located where eggshell mineralization occurs (Gautron et al., 2007). This protein is detected in uterine fluid and throughout the entire eggshell, especially at the inner part of the shell and at the mammillary layer (Gautron et al., 2007; Mikšík et al., 2007). Purification of OCX-36 revealed its antimicrobial activity against *Staphylococcus aureus*, and ability to bind lipopolysaccharide (LPS) from *Escherichia coli* and *S. aureus* lipoteichoic acid (LTA) (Cordeiro et al., 2013). These results support the proposed involvement of OCX-36 in the innate immune response, similar to other homologous members of the BPI/LBP/PLUNC family (Gautron et al., 2007, 2011). The OCX-36 protein sequence is composed of two lipid-binding domains BPI1 (BPI/LBP/CETP N-terminal domain) and BPI2 (BPI/LBP/CETP C-terminal domain) of about 200 amino acids each (Supplementary Figure 1) (Gautron et al., 2007). OCX-36 was initially thought to be eggshell-specific since this protein was first identified in chicken eggshell membranes and eggshell organic matrix. However, in addition to the distal oviduct, it is also expressed in the chicken intestine (Gautron et al., 2007; Tian et al., 2010; Chiang et al., 2011).

**FIGURE 5:**
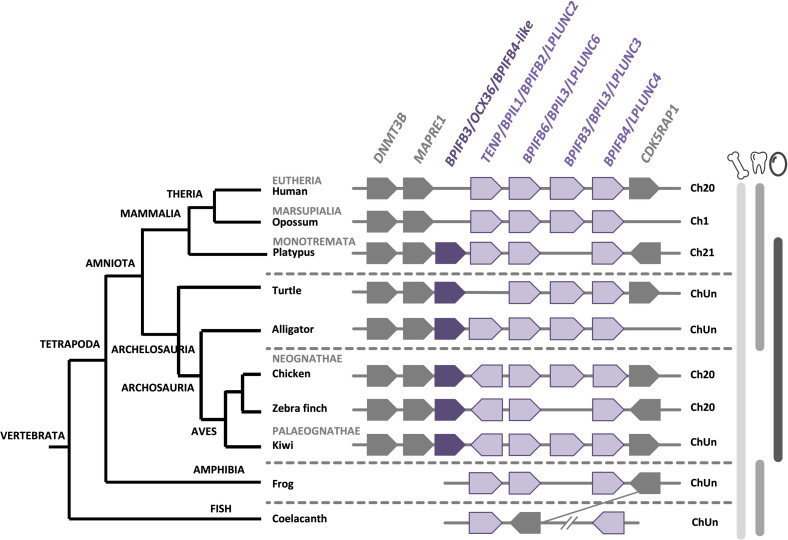
Synteny of *BPIFB3/OCX36* genes in birds and its relatives in vertebrates. The *BPIFB3/OCX36* gene is represented by a dark purple box and its relatives are represented by light purple boxes. Flanking genes DNA (cytosine-5)-methyltransferase 3B (*DNMT3B*), Microtubule-associated protein RP/EB family member 1 (*MAPRE1*) and Mitochondrial tRNA methylthiotransferase (*CDK5RAP1*) are represented by gray boxes. ChUn, chromosome unknown. Gene IDs are listed in Supplementary Table 2. The left part of the figure depicts the phylogenetic relationship between vertebrate species (adapted from www.tolweb.org and Kapusta et al., 2017).

**FIGURE 6:**
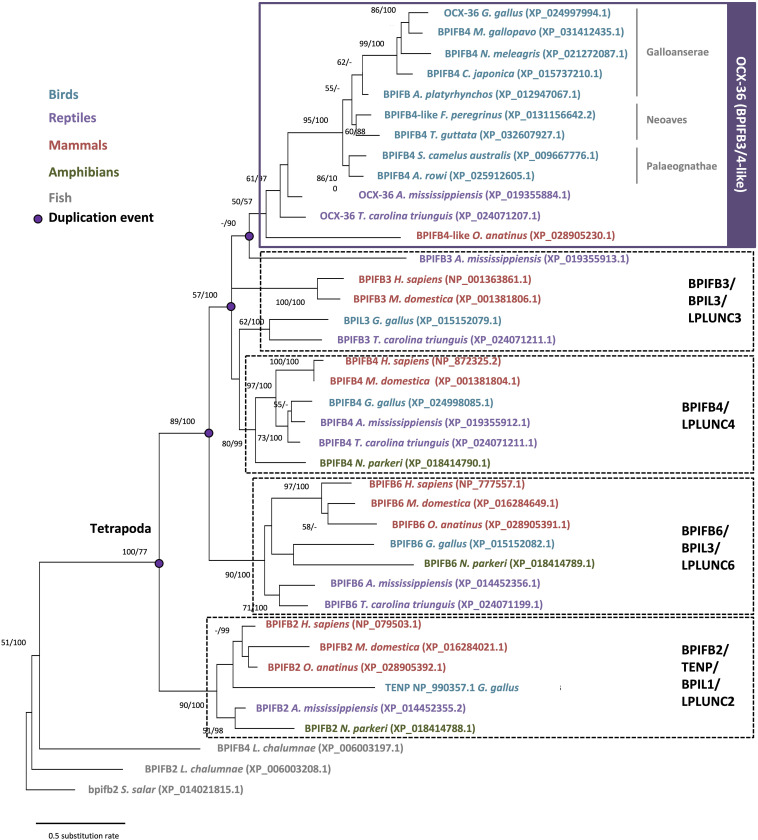
Phylogenetic reconstruction of OCX-36 and its orthologs and paralogs in vertebrates. For phylogeny multiple alignment was performed using ClustalW and Gblocks (www.phylogeny.fr). Model of protein evolution used is JTT + G (MEGAX v10.1.8; https://www.megasoftware.net/). Topology of the tree corresponds to maximum likelihood method with 100 repetitions (MEGAX v10.1.8). Bootstrap values from maximum likelihood/Bayesian inference are indicated at each node when value is >50.

The BPI/LBP/PLUNC protein family belongs to the TULIP (tubular lipid-binding) superfamily, which split into two groups before the last eukaryote common ancestor: SMP-like proteins (synaptotagmin-like, mitochondrial and lipid-binding proteins) and BPI-like proteins (Alva and Lupas, 2016). The BPI/LBP/PLUNC family is only present in animals (Alva and Lupas, 2016). Indeed, members of this gene family are found in both vertebrate and invertebrate species (Solstad et al., 2007; Chiang et al., 2011; Krasity et al., 2011). In vertebrates, although 20–30% of amino acid identity was observed between chicken OCX-36 and other BPI family B proteins (also called LPLUNCs) (Supplementary Table 1), the similar organization of exons/introns in members of this gene family strongly suggests a common origin by multiple duplication events (Gautron et al., 2007; Tian et al., 2010). Synteny analysis of this gene family confirms a common ancestor for the genes encoding chicken OCX-36, *BPIFB3* (gene synonym: *OCX36*) and other *BPI family B* members (Tian et al., 2010; Gautron et al., 2011) (Figure 5 and Supplementary Table 1). Since these previous analyses were performed, substantial new genomic and transcriptomic data has enriched this story. The synteny presented in Figure 5 shows the presence of a *BPIFB3*/*OCX36* orthologous gene in reptiles (turtle and alligator), and other bird species from Palaeognathae (kiwi), Neoaves (zebra finch), and Galloanserae (duck). In addition, analysis of the platypus genome (*Ornithorhynchus anatinus*), an egg-laying mammal (Monotremata), reveals the presence of *BPIFB4-like* gene at the same location as *BPIFB3/OCX36* in birds and reptiles (Figure 5). Identity and similarity levels are higher between platypus BPIFB4-like and chicken OCX-36 than between chicken OCX-36 and other chicken BPIFB paralogs (Supplementary Table 1), suggesting that platypus BPIFB4-like is the ortholog of avian OCX-36. Phylogenetic analysis of OCX-36 and its relatives shows a first cluster containing chicken OCX-36, BPIFB4-like from other birds, OCX-36-like from reptiles and BPIFB4-like from platypus (Figure 6). This cluster, in addition to the results of synteny analysis, strongly suggests that all these genes are orthologs of chicken OCX-36. These new inputs expand the presence of *BPIFB3/OCX36* orthologous genes to Archelosauria (turtles, crocodiles, and birds) and Monotremata (egg-laying mammals) phyla. Finally, these new insights invalidate the previous hypothesis that *BPIFB3*/*OCX36* arose after the divergence of birds and mammals (Tian et al., 2010; Chiang et al., 2011; Gautron et al., 2011). The phylogenetic tree coupled with synteny strongly support that *BPIFB3*/*OCX36* appeared before the divergence of birds and mammals, which was likely lost in therian mammals (placentals and marsupials) (Figures 5, 6). This phylogeny indicates that another member, *TENP* (transiently expressed in neural precursors), is the oldest gene in the BPI/LBP/LPLUNC family, and that the *BPIFB3/OCX36* gene is the result of three duplication events before tetrapod diversification and one event in amniotes.

New support for the specificity of OCX-36 protein orthologs to eggshell should be sought by investigation of diverse eggshell proteomes such as ratites, reptiles and monotremes. In the shell proteome of a crocodilian egg, an OCX-36 ortholog was not identified, but, as in birds, its paralog TENP-like is present in the shell organic matrix (Mann et al., 2006; Mikšík et al., 2018). In chicken, TENP is also found in egg white, vitelline membranes and egg yolk (Guérin-Dubiard et al., 2006; Mann, 2007; D’Ambrosio et al., 2008; Mann and Mann, 2008; Farinazzo et al., 2009). Apparently, TENP was recruited to the egg immune system in birds, while mammalian orthologs, BPIL1/BPIFB2/LPLUNC2, were recruited in ORL (olfactory epithelium, larynx, and tongue) tissue immunity (Andrault et al., 2003).

### Co-option of SIBLING Bone Proteins, Osteopontin (OPN/SPP1) and Ovocleidin-116 (OC-116/MEPE) in Eggshell Biomineralization

OPN is a phosphoprotein (SPP1, secreted phosphoprotein 1, is the mammalian ortholog) found in both avian bone and eggshell, as well as a variety of other tissue and cell types (Moore et al., 1991; Pines et al., 1995; Sodek et al., 2000; Fernandez et al., 2003; Chien et al., 2009; Hincke et al., 2012). In mice, OPN is strongly implicated in bone remodeling and fracture healing (McKee et al., 2011). In chicken, the oviduct expression of the OPN gene (*SPP1*) is entirely uterine-specific and is temporally associated with eggshell calcification through mechano-transcriptomic coupling of physical distension of the uterine wall to *SPP1* expression (Pines et al., 1995; Lavelin et al., 1998). Moreover, unusual patterns of uterine *SPP1* expression are associated with eggshell mineralization defects (Arazi et al., 2009). Localization by colloidal gold immunocytochemistry shows that OPN is concentrated in the palisade layer of the eggshell, where it is associated with parallel protein sheets of organic matrix, and more diffusely with the (104) crystallographic faces of eggshell calcite (Chien et al., 2008, 2009; Hincke et al., 2008). Specific OPN binding to the growing (104) crystal face during mineralization could modify the resistance of the shell to fracture along this plane. A functional interaction between OPN and the (104) eggshell calcite faces was supported by *in vitro* studies where synthetic calcite crystal growth at the (104) face was inhibited by added OPN (Chien et al., 2008). Nanoindentation and atomic force microscopy measurements suggest that OPN influences eggshell hardness and nanostructure, which in turn control the mechanical properties of the shell (Athanasiadou et al., 2018). SNPs in chicken *SPP1* are associated with eggshell fracture toughness (Dunn et al., 2009; Romé and Le Roy, 2016), which supports a regulatory role for OPN in mineralization. From fishes to mammals, OPN possesses a poly-aspartate motif, which is able to bind calcium and mediates binding to the mineral surface (Supplementary Figure 2; Sodek et al., 2000; Athanasiadou et al., 2018; Weber, 2018). However, in birds and reptiles, the OPN protein sequence exhibits a unique feature, a histidine-rich region that is suspected to originate from a microbial gene *via* a horizontal gene transfer event in early reptiles (Supplementary Figure 2; Weber, 2018). In mollusk shell, perlinhibin is a histidine-rich protein that inhibits calcium carbonate crystallization (Mann et al., 2007b), suggesting that this motif in eggshell OPN could play a similar calcite-specific role. In addition to the histidine-rich region, the C-terminal region is different between reptiles and non-reptilian vertebrates. In reptiles, the C-terminus is highly conserved, which supports a specialization of this protein with an important functional role in this vertebrate group (Supplementary Figure 2).

**FIGURE 7:**
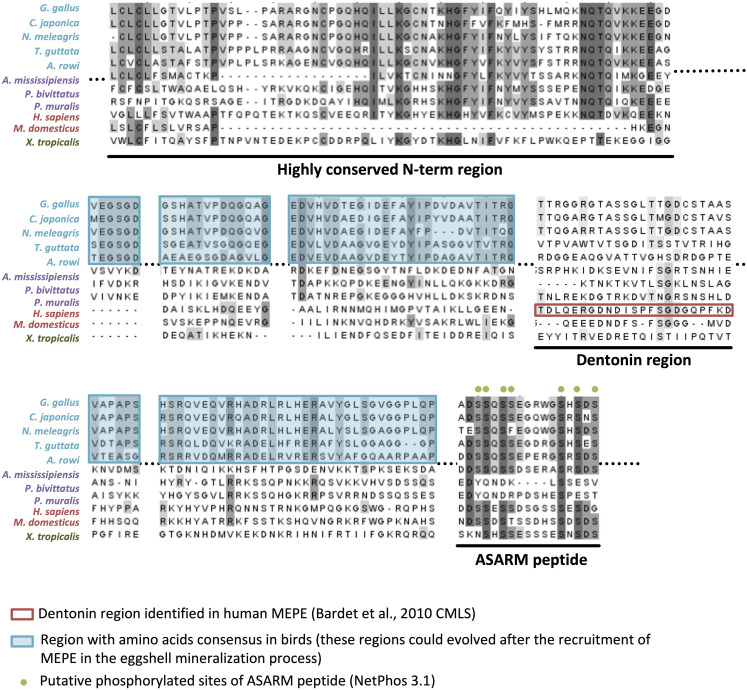
Multiple alignment of OC-116/MEPE proteins in tetrapods. The alignment was built with ClustalW (www.phylogeny.fr) and edited with Jalview v2.11.1.4 (https://www.jalview.org/). Gray colors of letters correspond to percentage of identity (dark gray > 80% identity, medium gray > 60% identity, and light gray > 40% identity). The dentonin region identified in human MEPE, and regions with amino acid consensus in birds, are, respectively, framed in red and blue. Putative phosphorylated sites of the ASARM (acidic, serine, and aspartic acid-rich motif) peptide are indicated by green dots above the alignment. Sequences aligned are from *Gallus gallus* (AAF00982.3), *Coturnix japonica* (XP_015716951.1), *Numida meleagris* (XP_021251779.1), *Taeniopygia guttata* (XP_030127798.1), *Apteryx rowi* (XP_025920573.1), *Alligator mississippiensis* (XP_019343676.1), *Python bivittatus* (XP_025029286.1), *Podarcis muralis* (XP_028598766.1), *Homo sapiens* (XP_006714341.1), *Monodelphis domestica* (XP_007495981.1), and *Xenopus tropicalis* (XP_002938672.2).

Ovocleidin-116 (OC-116; MEPE, matrix extracellular phosphoglycoprotein, is the mammalian ortholog) is a major component of the chicken uterine fluid, and is the most abundant matrix protein in the eggshell (Hincke et al., 1999; Mann et al., 2002, 2006; Marie et al., 2015a). It is an eggshell dermatan sulfate proteoglycan, which also possesses two *N*-glycosylated sites as well as *N*-glycan structures with fucosylated LacdiNAc (Nimtz et al., 2004). Immunostaining of the decalcified eggshell demonstrated the presence of OC-116 throughout the palisade layer and in the mammillary cone layer (Hincke et al., 1999). OC-116 is present in both soluble and insoluble fractions of the chicken eggshell matrix (Mann et al., 2002, 2006; Mikšík et al., 2007). Proteomics studies have identified OC-116 in the eggshell of chicken, turkey, quail, mallard duck and Guinea fowl, where it is one of the most abundant eggshell constituent (Mann and Mann, 2013, 2015; Marie et al., 2015a; Le Roy et al., 2019; Zhu et al., 2019). In addition to the eggshell, OC-116 was reported in the chicken cortical and medullary bone (Horvat-Gordon et al., 2008). SNP analysis revealed that the *MEPE/OC116* gene is associated with shell thickness, elastic modulus and egg shape (Dunn et al., 2009; Romé and Le Roy, 2016). The mammalian ortholog MEPE is involved in bone and teeth mineralization (Bardet et al., 2010a). Inactivation of the *Mepe* gene in mouse causes an increase in bone mass and mineralization due to an increase in osteoblast number and activity (Gowen et al., 2003). The role of OC-116/MEPE in mineralization is supported by the ASARM (acidic serine-aspartate rich MEPE-associated motif) sequence located at the C-terminus of the protein. When ASARM is phosphorylated, it inhibits mineralization by binding to hydroxyapatite crystals (Addison et al., 2008). This peptide is also involved in phosphatemia regulation (Rowe et al., 2004). Multiple sequence alignment of OC-116/MEPE proteins shows a high conservation of the ASARM peptide throughout tetrapods (Figure 7). The presence of numerous putative phosphorylation sites (7 in bird orthologs) suggests that its role in mineralization is also conserved in both bone and eggshell.

**FIGURE 8:**
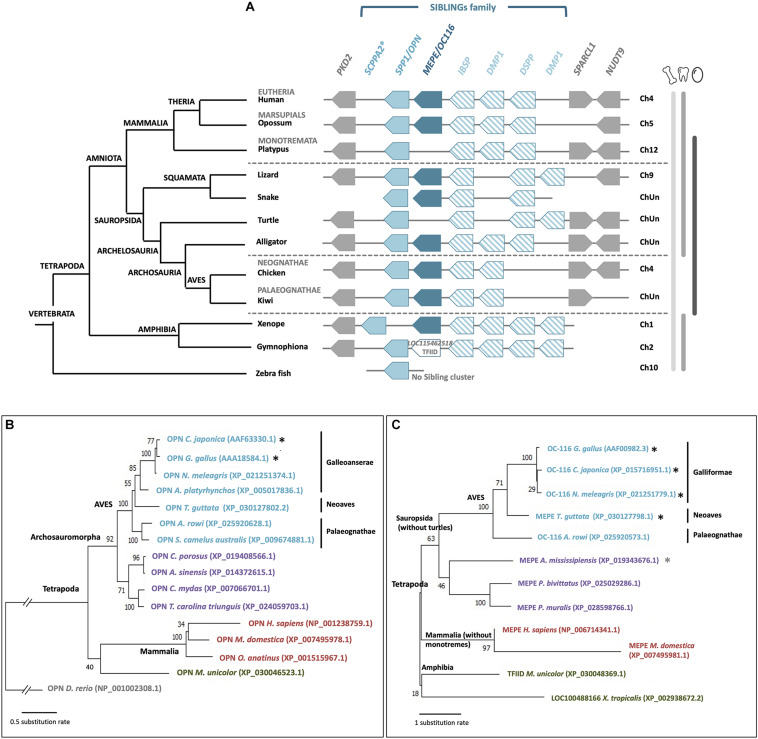
Synteny and phylogeny of *OPN/SPP1* and *MEPE/OC116* genes and corresponding proteins in vertebrates. **(A)** *OPN/SPP1* is represented by a light blue box and *MEPE/OC116* is represented by a dark blue box. The other SIBLING genes are integrin-binding sialoprotein (*IBSP*), dentin sialophosphoprotein (*DSPP*) and dentin matrix protein 1 (*DMP1*), which are represented by empty boxes with oblique blue lines. In Gymnophiona (amphibian), instead of *MEPE* we observed the presence of the transcription initiation factor TFIID subunit 1-like gene (*LOC115462518*, XP_030048369.1). Flanking genes are polycystin 2 (*PKD2*), SPARC-like 1 (*SPARCL1*) and nudix hydrolase 9 (*NUDT9*), which are represented by gray boxes. ChUn: Chromosome Unknown. Blue asterisk indicates that *SCPPA2* is a hypothetical ortholog of *spp1* (Kawasaki, 2011). Gene IDs are listed in Supplementary Table 2. In the left part, the phylogenetic tree for vertebrate species is represented (adapted from www.tolweb.org and Kapusta et al., 2017). **(B)** Phylogeny of OPN/SPP1 in vertebrates, which was reconstructed using multiple alignment performed with ClustalW and Gblocks (www.phylogeny.fr) and JTT + G model of protein evolution (MEGAX v10.1.8; https://www.megasoftware.net/). Topology of the tree corresponds to maximum likelihood method (MEGAX v10.1.8). Bootstrap values from maximum likelihood are indicated at each node. The symbol//indicates a gap of 0.8 substitution rate to add to the basal branch. Black asterisks indicate species where the protein was identified in the eggshell. **(C)** Phylogenetic reconstruction of OC-116/MEPE in vertebrates was performed using multiple alignment constructed with ClustalW and Gblocks (www.phylogeny.fr) and JTT + G + F model of protein evolution (MEGAX v10.1.8). Topology of the tree corresponds to maximum likelihood method (MEGAX v10.1.8). Bootstrap values from maximum likelihood are indicated at each node. Black asterisks indicate species where the protein was identified in the eggshell and the gray asterisk indicates a related species (*C. siamensis*) where the protein was identified in the eggshell.

Both OPN/SPP1 and OC-116/MEPE belong to the SIBLING (small integrin-binding ligand N-linked glycoprotein) family, with three other protein members: integrin-binding sialoprotein (IBSP), dentin sialophosphoprotein (DSPP) and dentin matrix protein 1 (DMP1). Genes coding for these five proteins are clustered together through tetrapods (Figure 8A), and they all have a role in biomineralization (Rowe, 2012); however, none of them appears to be specific to calcium carbonate (eggshell) or calcium phosphate (bone, teeth) mineralization. They possess similar molecular properties such as integrin-binding and calcium-binding (Bardet et al., 2010b). Among the five SIBLING members, OPN and OC-116 have been widely studied in the chicken eggshell, as described above. Moreover, DMP1 and IBSP were detected in the eggshell matrix (by proteomics and Western blotting), and their genes are expressed in uterine tissue (Mann et al., 2006; Horvat-Gordon et al., 2008); however, *DSPP* gene, involved in dentin formation, was secondarily lost in ancestors of birds during late Cretaceous when they become toothless (Figure 8A) (Kawasaki and Weiss, 2008; Sire et al., 2008; Mcknight and Fisher, 2009; Kawasaki, 2011; Sire and Kawasaki, 2012). It has been suggested that the entire SIBLING gene family, including OC-116/MEPE and OPN/SPP1, which were initially involved in bone formation, was co-opted for the eggshell calcium carbonate mineralization process in birds (Sire and Kawasaki, 2012). In the phylogenetic reconstruction of OC-116 and OPN, the distribution of both proteins follows the accepted phylogenetic relationships in tetrapods, with a clear split between mammals and reptiles including birds (Figures 8B,C). In the Aves clade, Palaeognathae is the basal group; while in Neognathae, Neoaves sequences are well separated from Galloanserae for OPN, and from Galliformae for OC-116. In the recent crocodilian eggshell proteome, OC-116 was identified but not OPN (Mikšík et al., 2018), suggesting the recruitment of OC-116, at least for eggshell mineralization, in the Archosauria linage (Aves and Crocodylia). In seven turtle species and platypus (*O. anatinus*, the only monotreme genome in NCBI database), although gene coding for SPP1 is present, synteny analysis reveals the absence of *MEPE/OC116* from the SIBLING locus (Figure 8A), which implies a loss of this gene in these two lineages.

### The Glycoprotein EDIL3: A Novel Candidate for Calcium Carbonate Delivery in Eggshell Mineralization

The glycoprotein EDIL3 (EGF-like repeats and discoidin domains 3) was identified in the chicken eggshell by proteomics analysis (Mann et al., 2006; Marie et al., 2015a). The EDIL3 sequence contains three EGF-like domains and two F5/8C (discoidin) domains; it was first identified as an extracellular matrix protein involved in embryonic vascular development in mouse (Hidai et al., 1998). The three EGF-like domains are present in EDIL3 orthologs in all vertebrates except in fishes (Stapane et al., 2019), and the third domain possesses a calcium-binding site, which suggests a potential role of EDIL3 in calcium carbonate crystallization (Marie et al., 2015a). In addition, an RGD (arginine, glycine, and aspartate) motif is present in the second EGF-like domain, through which it can bind integrins (Stapane et al., 2019). Integrins are transmembrane proteins involved in cell-cell and cell-extracellular matrix interactions, and vesicular trafficking (Théry et al., 1999; Hynes, 2002; Gatti et al., 2005; Bridgewater et al., 2012). The second F5/8C domain exhibits a phospholipid-binding site, which give to the protein the ability to complex vesicle and/or cell membranes (Supplementary Figure 3A; Stapane et al., 2019). In the EDIL3 sequence of some bird species (*G. gallus*, *A. platyrhynchos*, *Aquila chrysaetos*, *Dromaius novaehollandiae*, etc.), the first EGF-like domain also contains an RGD motif suggesting an even higher affinity of the protein for integrins (Supplementary Figure 3B). Proteomics demonstrate the presence of EDIL3 in bird and crocodile eggshell (Marie et al., 2015a; Mikšík et al., 2018; Le Roy et al., 2019). However, in the eggshell proteome of *Crocodylus siamensis*, EDIL3 protein was identified with six peptides matching with EDIL3 from *Alligator mississippiensis* (NCBI accession KYO21076.1) (Mikšík et al., 2018). Surprisingly, amongst the six peptides, only one matched with a domain present in EDIL3 proteins (first F5/8C domain); however, the five other peptides matched with the IG-like (immunoglobulin-like) and LINK_2 domain (hyaluronan-binding region) that is found in HAPLN1 (hyaluronan and proteoglycan link protein 1). Moreover, the annotated *A. mississippiensis* EDIL3 protein is 843 aa in length instead of about 480 aa for the other EDIL3 proteins, and seems to be composed of both EDIL3 and HAPLN1 protein features (Supplementary Figure 3C). Indeed, the N-terminal part of alligator EDIL3 shows 53.8% of identity with chicken EDIL3 and the C-terminal part of alligator EDIL3 possesses 54.3% of identity with chicken HAPLN1. These contradictions indicate that the potential identification of the EDIL3 ortholog in crocodilian eggshell requires confirmation (Supplementary Figure 3). In chicken, EDIL3 is not an eggshell-specific protein, although it exhibits a high relative abundance in the shell OM (Marie et al., 2015a). According to the emPAI (exponentially modified protein abundance index) values of proteins from chicken eggshell proteome at four calcification stages, EDIL3 is the fifth most abundant protein at the early stages of biomineralization, corresponding to the transformation of ACC into calcite crystals (Marie et al., 2015a).

In chicken, *EDIL3* gene expression is up-regulated in isthmus and uterus compared with bone, duodenum, kidney, liver and magnum, and is significantly higher in the oviduct segments at early stages (6 and 7 h post-ovulation, initiation of mineralization) than at 16 h post-ovulation (mid-calcification) (Stapane et al., 2019, 2020). Immunohistochemistry in uterine cross-sections confirms the presence of high levels of EDIL3 at the early stages of mineralization in tubular gland cells (5 and 6 h post-ovulation) (Stapane et al., 2020). Moreover, proteomics and Western blot analyses revealed the presence of EDIL3 in extracellular vesicles isolated from chicken uterine fluid (Stapane et al., 2019, 2020). These vesicles are proposed to mediate the transportation of ACC to the mineralization site. Indeed, vesicles have been demonstrated to play roles in ACC stabilization in invertebrate biomineralization models such as sea urchins, molluscan shell and coral skeleton (Levi-Kalisman et al., 2002; Addadi et al., 2003; Weiner and Dove, 2003; Mass et al., 2017). In chicken uterus samples examined by transmission electron microscopy, extracellular vesicles are observed in uterine cells and fluid, and vesicles are seen budding from cells into the uterine lumen. Energy-dispersive electron spectroscopy and selected area electron diffraction revealed the presence of ACC in extracellular vesicles purified from the uterine fluid (Stapane et al., 2020). Based on these and other results, extracellular vesicles are proposed to play a role in ACC-mediated calcification of the eggshell. EDIL3 is proposed to bind vesicle membrane (phospholipid-binding site/integrin-binding site) and to guide these vesicles from uterine cell cytosol to the mineralization site (calcium-binding site/integrin-binding site) in the uterine fluid of chicken (Stapane et al., 2019, 2020).

The gene *EDIL3* is highly conserved in vertebrates (Supplementary Figure 4) (Stapane et al., 2019). The flanking genes in this locus are versican (*VCAN*) and hyaluronan and proteoglycan link protein 1 (*HAPLN1*). Phylogenetic analysis of EDIL3 and its paralog MFGE8 in animals demonstrates the appearance of both proteins after a duplication event in vertebrates 480 MYA (Stapane et al., 2019). EDIL3 was subsequently recruited to the eggshell mineralization process, at least in the Aves phylum. Interestingly, its paralog MFGE8 is also detected in the eggshell proteome of birds; however, its abundance is much lower than EDIL3, and *MFGE8* expression is not specific to tissues responsible for eggshell mineralization or to the initial stages of mineralization (Stapane et al., 2019).

## A Protein Specific to the Eggshell Biomineralization Process

### C-Type Lectin Proteins in Eggshell Organic Matrix: Ovocleidin-17 Homologs

The C-type lectin protein Ovocleidin-17 (OC-17) is an eggshell-specific protein, which was first purified and partially sequenced from the chicken eggshell (Hincke et al., 1995). The mRNA sequence was determined only recently by *de novo* transcriptomic assembly (Zhang et al., 2014). OC-17 contains a C-type lectin (CTL) domain and possesses two phosphorylated serine residues (Mann, 1999; Mann and Siedler, 1999). The CTL proteins are a huge family of proteins including at least seven subgroups such as hyalectans, asialoglycoprotein receptors, collectins, selectins, natural killer group transmembrane receptors, macrophage mannose receptors and simple lectins (Zelensky and Gready, 2005). OC-17 and its homologs correspond to a simple lectin, with a short amino acid sequence (about 150 aa) and only one CTL domain. Proteomics analysis demonstrated that OC-17 is a highly abundant protein in the eggshell matrix in chicken and Guinea fowl (Marie et al., 2015a; Le Roy et al., 2019). Moreover, CTL proteins that are homologs of OC-17 have been identified in eggshells of many bird species, including ostrich, emu, and rhea (Mann and Siedler, 2004, 2006). In each of these ratites, two homologous CTL eggshell proteins were identified and named according to the bird species: Struthiocalcin-1 and 2 (SCA-1 and -2) for ostrich, Dromaiocalcin-1 and -2 (DCA-1 and -2) for emu and Rheacalcin-1 and -2 (RCA-1 and -2) for rhea. For easier reading in the present review, we have termed these proteins XCA-1 and XCA-2. In contrast, only one CTL protein (OC-17) is present in chicken eggshell, which aligns with the XCA-2 group of other bird species.

C-type lectin proteins have been identified in the biomineralization process of invertebrates. For instance, in the sea urchin *Strongylocentrotus purpuratus*, SM50 is a protein containing a C-type lectin domain in addition to glycine-rich and proline-rich regions. The study of this C-type lectin domain revealed that it influences the biomineralization of CaCO_3_ (Rao et al., 2013). In the same manner, in the freshwater pearl mussel, a C-type lectin protein called perlucin, already identified in the shell proteomes of mollusks, is involved in nacre formation (Lin et al., 2013). Purified OC-17 modifies calcite crystallization *in vitro* (Reyes-Grajeda et al., 2004). *In silico* molecular dynamics simulations suggest three protein configurations of OC-17, which is able to bind calcium carbonate surfaces through its positively charged guanidino group of specific arginine residues (Freeman et al., 2010, 2011). Thus, CTL proteins could play a role in eggshell formation by binding to specific calcite crystal faces (Wallace and Schiffbauer, 2016). In addition, chicken OC-17 and its goose ortholog (ansocalcin) exhibit an antimicrobial activity, and could play a potential role in innate immunity of the avian embryo (Wellman-Labadie et al., 2008a). Although the presence of one versus two OC-17 paralogs in the eggshell of Palaeognathae birds has been proposed to correlate with eggshell thickness (Mann and Mann, 2015), there is currently no experimental evidence to support this hypothesis.

**FIGURE 9:**
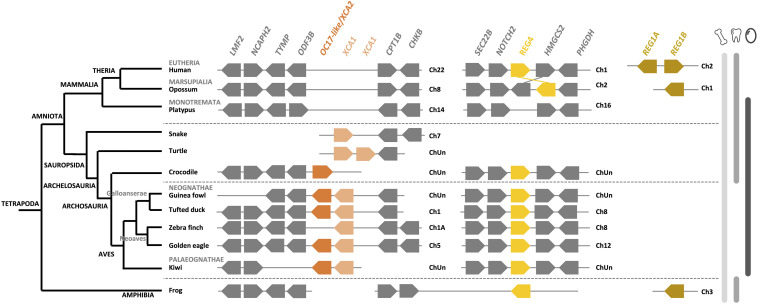
Synteny of *OC17-like*, *XCA* and *REG* orthologs and paralogs in birds and its relatives in vertebrates. *OC17-like/XCA2* is represented by a dark orange box and *XCA1* by a light orange box. *REG4* (regenerating Islet-derived protein 4) is represented by a yellow box and *REG1 A* and *B* (Lithostathine) are represented by a brown box. Flanking genes are represented by gray boxes: lipase maturation factor 2 (*LMF2*), condensing-2 complex subunit H2 (*NCAPH2*), thymidine phosphorylase (*TYMP*), outer dense fiber protein 3B (*ODF3B*), carnitine *O*-palmitoyltransferase 1 (*CPT1B*), choline/ethanolamine kinase (*CHKB*), vesicle-trafficking protein (*SCE22B*), neurogenic locus notch homolog protein 2 (*NOTCH2*), hydroxymethylglutaryl-CoA synthase (*HMGCS2*) and D-3-phosphoglycerate dehydrogenase (*PHGDH*). Gene IDs are listed in Supplementary Table 2. The left part of the figure depicts the phylogenetic relationship between vertebrate species (adapted from www.tolweb.org and Kapusta et al., 2017).

**FIGURE 10:**
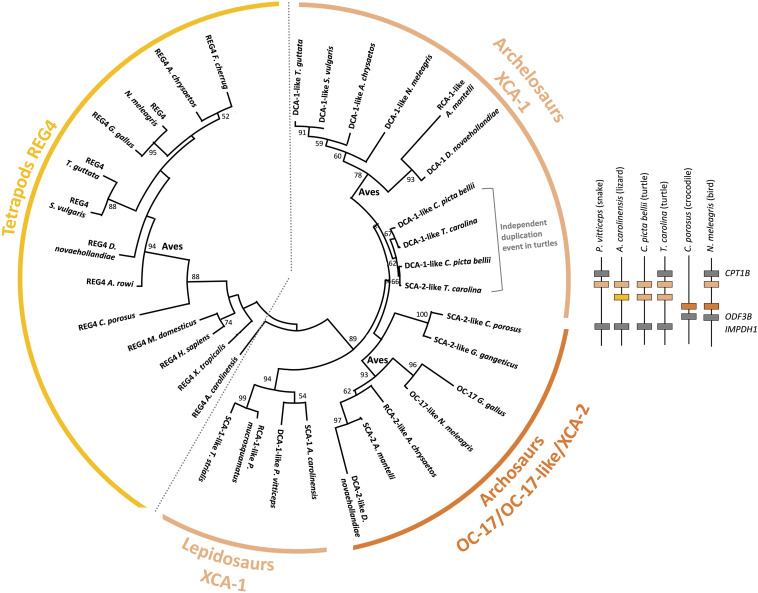
Phylogenetic reconstruction of OC-17 and its orthologs and paralogs in vertebrates. Phylogeny was performed using the maximum likelihood method with 100 repetitions (MEGAX v10.1.8; https://www.megasoftware.net/) using ClustalW multiple alignment and Gblocks (www.phylogeny.fr), and the WAG + G model of protein evolution. Bootstrap values from maximum likelihood are indicated at each node when value is >50. Synteny of *OC-17-like/XCA-2* and *XCA-1* genes is represented for five reptilian species (*Pogona vitticeps*, *Anolis carolinensis*, *Chrysemys picta bellii*, *Terrapene carolina triunguis*, and *Crocodylus porosus*) and one bird (*Numida meleagris*).

Synteny, protein multiple alignment and phylogenetic analysis of XCA-1, XCA-2 and OC-17-like confirm that XCA-2 is ortholog to OC-17-like and XCA-1 is paralog to OC-17-like/XCA-2 (Figures 9, 10, and Supplementary Figure 5). OC-17-like and XCAs are also similar to other vertebrate C-type lectin (CTL) proteins, such as REG4 (Regenerating Islet-derived protein 4) and Lithostathine (also known as REG1A and REG1B) in humans. REG1 is a pancreatic CTL protein involved in the inhibition of CaCO_3_ precipitation in the bicarbonate-rich pancreatic juice (Bernard et al., 1992). The gene encoding REG1/Lithostathine is not found in the genome of reptiles and birds, whereas *REG4* is present in numerous bird and crocodilian species. The OC-17-like/XCAs gene symbol in reptiles and birds is different for each species (i.e., *LOC numbers*; Supplementary Table 3); in order to simplify our discussion, we use the arbitrary nomenclature *OC17-like/XCA2* and *XCA1*. Synteny analysis shows that the *REG4* gene is located on a different chromosome than the *OC17-like/XCA2* and *XCA1* gene locus (e.g., in tufted duck *REG4* is located on chromosome 8 and *OC17-like/XCA2* and *XCA1* are located on chromosome 1; Figure 9). *REG4* is flanked by the same genes in crocodiles, birds and in mammals, but it is absent in turtles and lepidosaurs (lizards, snakes, etc.) at the same locus. On the other hand, *OC17-like/XCA2* and/or *XCA1* genes are clustered together and flanked by the same genes in birds and reptiles; however, they are absent from the same locus in mammalian and amphibian genomes. These data support the hypothesis that OC-17-like/XCA-2 and XCA-1 are eggshell specific proteins and that these genes are specific to vertebrates that produce a calcitic shell; however, no ortholog has yet been identified in the crocodilian eggshell proteome (Mikšík et al., 2018). Deeper investigation needs to be done in crocodilian eggshells, but also in other reptile eggshells (e.g., snakes, lizards, and turtles), in order to determine if OC-17-like/XCA-2 and/or XCA-1 are widespread in reptile and bird eggshells or if they strictly correspond to the bird eggshell biomineralization process.

The pairwise alignment of chicken REG4 and OC-17 amino acid sequences exhibits 29.2% identity (58.4% similarity), which supports a common origin of both proteins (Supplementary Table 4). Phylogenetic reconstruction shows that REG4 and OC-17-like/XCAs are divided into two distinct groups (Figure 10). Regarding these observations, the phylogeny indicates that OC-17-like/XCAs arose from a duplication event in Sauropsida. In this clade, squamates (lizards and snakes) are in basal position with only one form of XCA. Then, three groups split: bird OC-17-like/XCA-2 (including crocodilian XCA-2), turtle XCA-1 and bird XCA-1 (Figure 10). The synteny of *OC17-like/XCA2* and *XCA1* shows that duplication of the ancestral gene occurred on the same chromosome and the phylogeny suggests that *XCA1* is closer to the ancestral form of the duplicated gene. These observations might indicate that *OC17-like/XCA2* is the result of a duplication event in archosaurs with a loss of *XCA1* in crocodilians. In turtles, two *XCA1* paralogs are also present but they are clustered together suggesting an independent duplication event in the turtle phylum.

In birds, XCA paralogs are present in both Neognathae (Neoaves and Galloanserae), and Palaeognathae (ratites) phyla. Nevertheless, in Neognathae, each bird species does not possess the two paralogs inside sub groups. In Neoaves we notice that only XCA-1 is present in common starling (*Sturnus vulgaris*), falcons (*Falco cherrug* and *Falco rusticolis*) and zebra finch (*Taeniopygia guttata*), whereas both paralogs are present in golden eagle (*A. chrysaetos chrysaetos*) (Supplementary Figure 6). In Galloanserae, some species exhibit one paralog such as chicken (*G. gallus*) and pheasant (*Phasianus colchicus*) (Mann et al., 2006; Marie et al., 2015a), for which the genome position is unknown. Conversely, other Galloanserae species possess both paralogs such as Guinea fowl, black swan (*Cygnus atratus*) and tufted duck (*Athya fuligula*) (Supplementary Figure 6) (Le Roy et al., 2019). The mallard duck eggshell proteome demonstrated the presence of an ortholog to chicken OC-17 protein (Zhu et al., 2019), which may correspond to mallard SCA-2-like protein translated from the newly submitted (December 2020) mallard genome in NCBI (Accession XP_038024161.1; Gene ID: 119713911). In this genome, the gene coding for SCA-2-like is located next to RCA-1-like (Gene ID: 119713283) in the same gene cluster containing *OC17-like/XCA2* and *XCA1*, as observed in other birds (Figure 9 and Supplementary Figure 6). In Palaeognathae, both paralogs are present in emu, ostrich and rhea eggshells, but in two *Apteryx* species (kiwis), for which eggshell proteomes are not available, one species exhibits two adjacent paralogs and the other species has only one paralog in its genome (Supplementary Figure 6). Nevertheless, the lack of genome data (gene sequencing and scaffolding genome assembly) is possibly the reason for the absence of the second paralog in all these species of birds, crocodilian etc. Indeed, the chicken OC-17 transcript has a very high GC content (72.17%), which could account for the observed difficulty to sequence this gene in the chicken genome and in genome projects of other bird species. Hence, this lack of *OC17-like/XCAs* annotation in bird genomes reduces the possibility to identify orthologous OC-17 peptides using proteomics approaches. This is the case in turkey and quail eggshell proteomes (Mann and Mann, 2013; Mann, 2015). In the Guinea fowl, OC-17-like (71.56% GC) and DCA-1-like (72.01% GC) were detected in its eggshell proteome, likely because the genome assembly of *Numida meleagris* that is available in the NCBI database (NumMel1.0) was built using the *G. gallus* genome.

## Evolution of Organic Matrix Proteins in Eggshell Biomineralization

**FIGURE 11:**
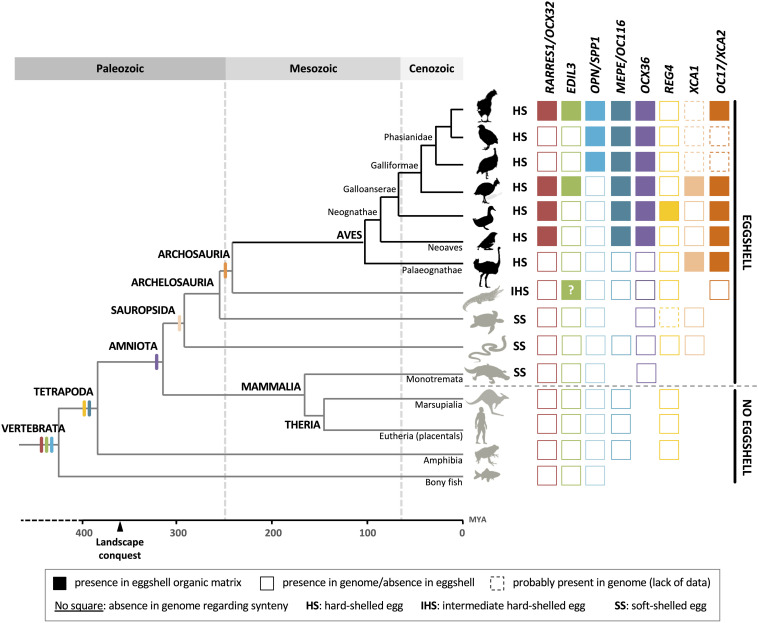
Schematic summary of the presence of genes encoding investigated proteins in vertebrate genomes and the identification of their related proteins in Sauropsida eggshells. Filled squares indicate the presence of the protein in eggshell organic matrix whereas empty squares indicate the presence in genome but the absence in eggshell. Squares with a dotted line indicate a putative presence in genome (lack of data). The absence of square in the figure illustrates an absence in genome with respect to synteny. The “?” in the crocodilian EDIL3 square indicates the uncertainty of true identification of this protein in the crocodilian eggshell proteome (see Supplementary Figure 3). Genomic data can come from different species inside the same apical group. The divergent times and topology of the tree are from (www.tolweb.org; Warren et al., 2008; Phillips et al., 2009; Shen et al., 2011; Jarvis et al., 2014; Kapusta et al., 2017).

During amniote evolution, reproduction was freed from reliance on the aquatic environment with the emergence of two possible reproductive strategies: egg-laying vs. placentation. In sauropsids and some mammals (Monotremata), the egg-laying strategy was based on a soft or hard-shelled egg to protect the embryo. This adaptation has reached its most advanced development in birds that emerged 102 MYA. The avian eggshell proteome exhibits both co-opted proteins and eggshell-specific proteins. Over the last several decades, the development of high-throughput technologies has helped to characterize and enumerate the complexity of the organic matrices in multiple avian species. The evolution of the eggshell reproductive strategy in sauropsids was accompanied by (1) the recruitment of existing genes for eggshell biomineralization (OCX-32, EDIL3, OC-116/MEPE, and OPN), and (2) the birth of new genes from duplication events, which are highly specialized for this process (XCA-1 and OCX-36; Figure 11). It is intriguing that avian OPN orthologs appear to have acquired a His-rich domain which may be related to calcitic biomineralization. Therefore, insight into the common eggshell toolkit which is responsible for eggshell mineralization in birds is emerging.

The huge outpouring of genomic data for bird species from the Bird 10,000 Genomes Project (2015–2020) is of vital importance to better understand the evolution of genes coding for eggshell proteins inside Aves (Palaeognathae vs. Neognathae; Neoaves vs. Galloanserae). In addition, this genomic database enrichment is crucial for future eggshell proteome studies in other bird groups (especially in Palaeognathae, for which only 2 proteins – the paralogs XCA-1 and XCA-2 – have yet been identified in the eggshell organic matrix). Finally, in order to enrich the evolutionary perspective, expanded analysis of eggshell proteomes should be performed in several reptilian species for which genomic/transcriptomic dataset are available, and in monotreme species such as platypus. Although the only crocodilian eggshell proteome exhibits common proteins with bird eggshell proteomes, it suffers from a very low number of identified proteins (58 against 904 for the chicken eggshell proteome), likely due to the absence of genomic/transcriptomic data for this species and incomplete annotation of genomic datasets of other crocodilians. And finally, the eggshell matrix databases must be enriched with post-translational modification (PTM) information, especially phosphorylation and glycosylation, which will permit cross-species comparisons for further insight. Currently, this information only exists in a comprehensive manner for the chicken eggshell phospho-proteome (Mann et al., 2007a).

This review identifies challenges and proposes new strategies to better understand the evolution of eggshell biomineralization, such as the multiplication of eggshell proteomics analyses in basal birds (ratites), sister groups of birds (crocodiles, turtles, squamates), and in more distant groups (monotremes).

## Author Contributions

MH and NL coordinated writing of the manuscript and edited the review. JG and LS contributed to the manuscript, wrote part of the review, and approved the final version. All authors contributed to the article and approved the submitted version.

## Conflict of Interest

The authors declare that the research was conducted in the absence of any commercial or financial relationships that could be construed as a potential conflict of interest.

## Footnotes

1 http://www.ensembl.org/index.html

2 https://b10k.genomics.cn/index.html
