# Supplementary figures and images for: Evolution of the Avian Eggshell Biomineralization Protein Toolkit – New Insights From Multi-Omics
Source: Front Genet. 2021 May 11;12:672433. doi: 10.3389/fgene.2021.672433 (PMC8144736; doi:10.3389/fgene.2021.672433)

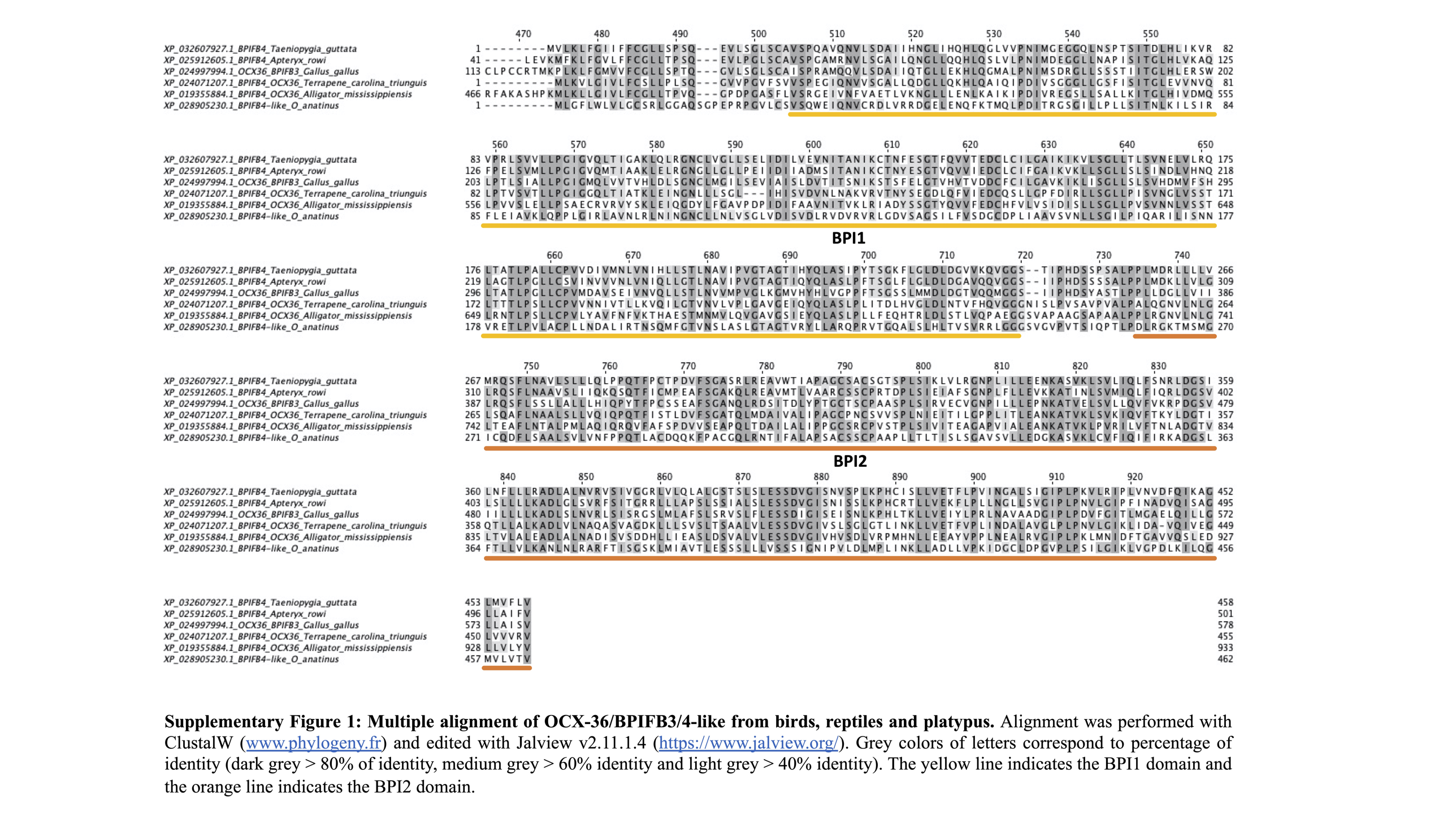

Supplement: Supplementary file 1 [file Image_1.jpg]

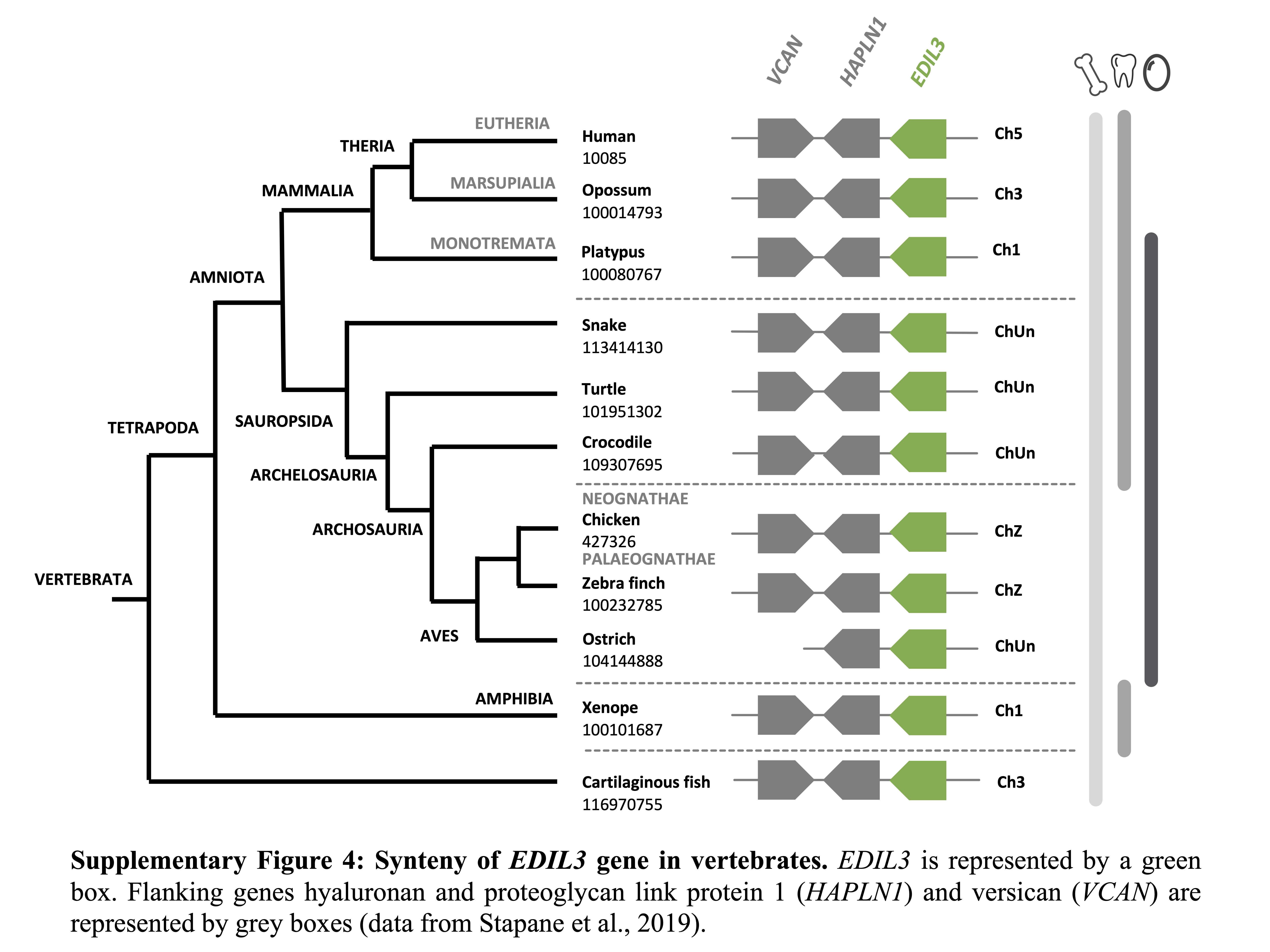

Supplement: Supplementary file 4 [file Image_4.jpg]

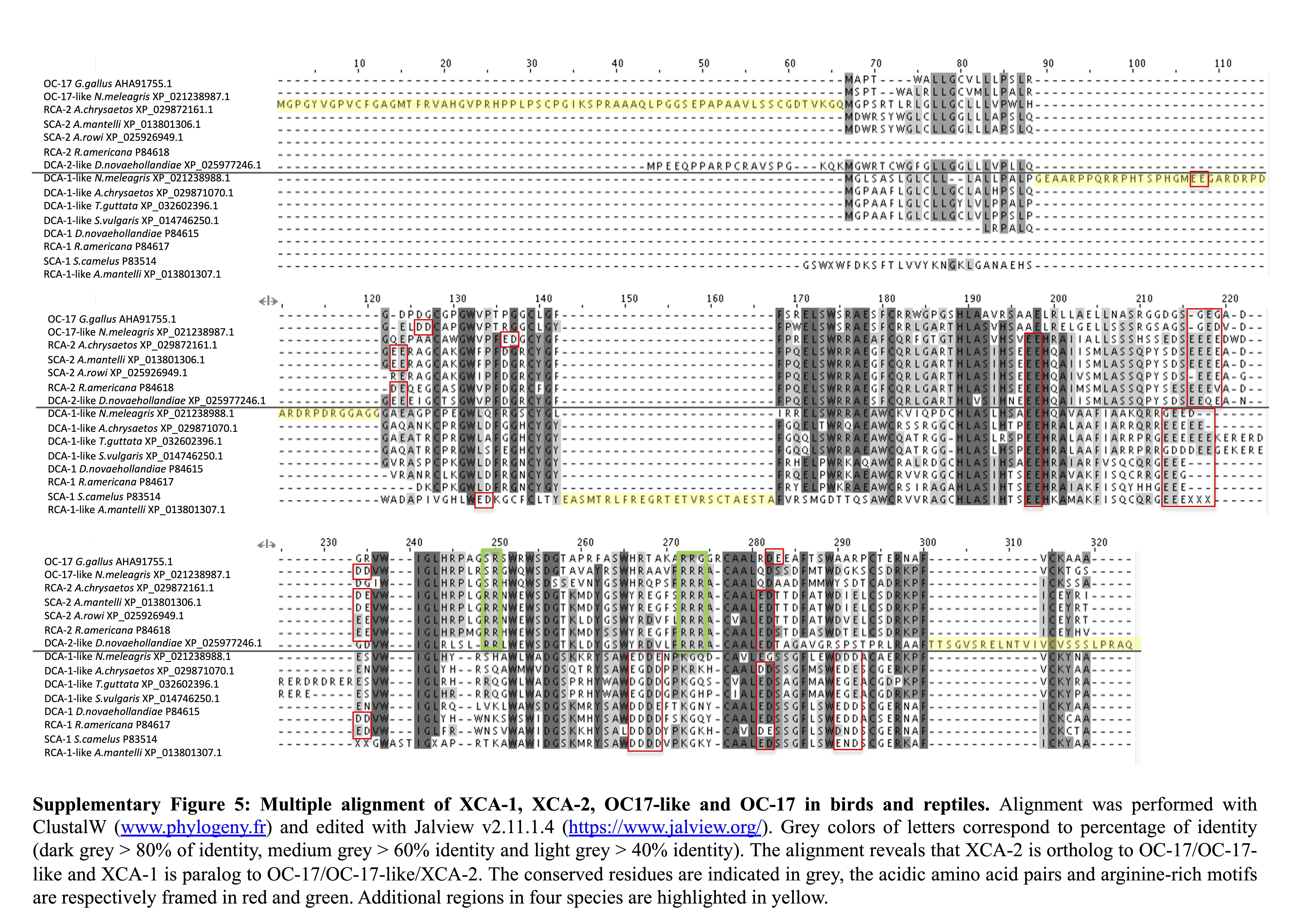

Supplement: Supplementary file 5 [file Image_5.jpg]

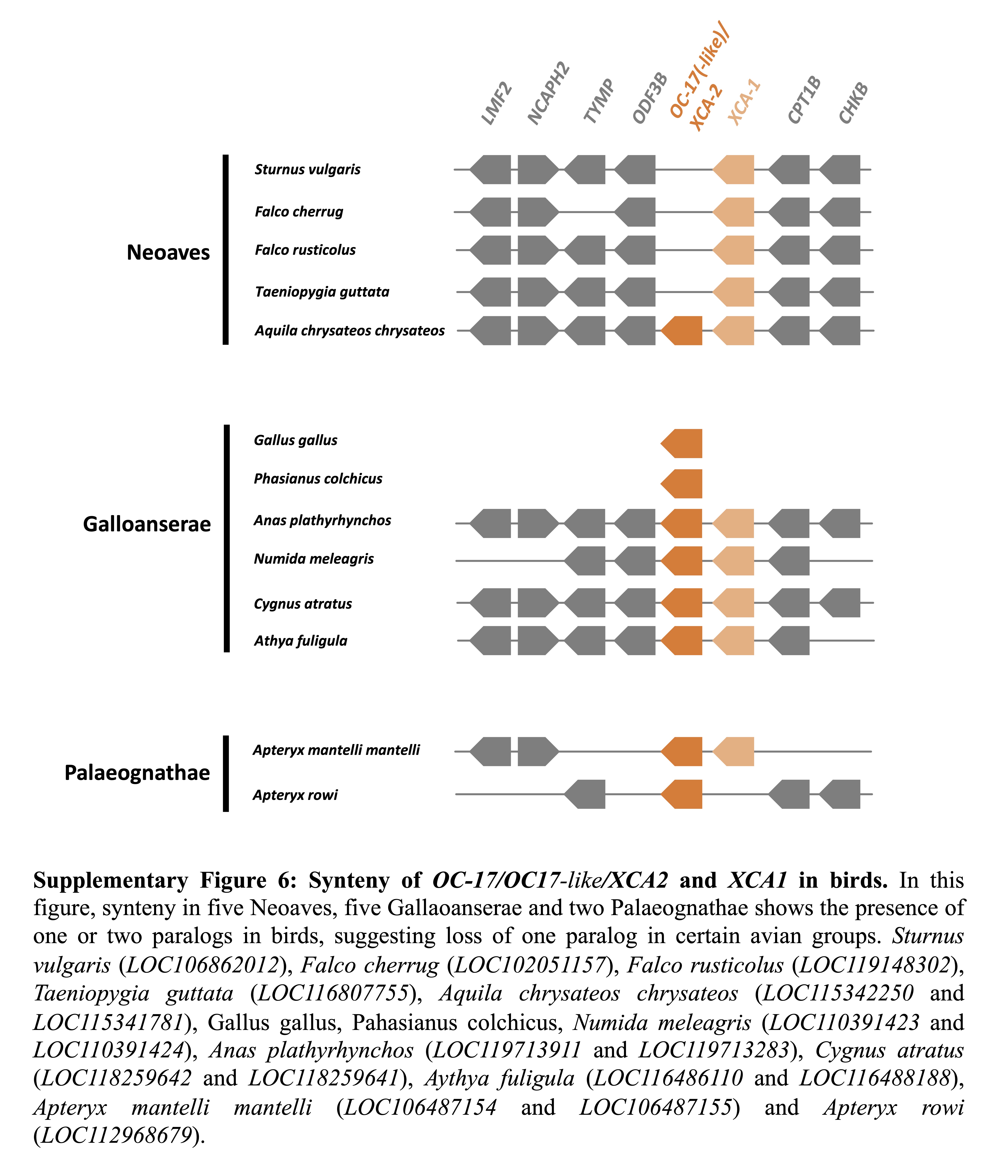

Supplement: Supplementary file 6 [file Image_6.jpg]
