# Supplementary material for: Evolution of the Avian Eggshell Biomineralization Protein Toolkit – New Insights From Multi-Omics
Source: Front Genet. 2021 May 11;12:672433. doi: 10.3389/fgene.2021.672433 (PMC8144736; doi:10.3389/fgene.2021.672433)
Supplement: Supplementary file 7 [file Table_1.DOCX]

**Supplementary Table 1:** **Identity and similarity between OCX-36 protein and its relatives in chicken and with BPIFB4-like protein of platypus (*Ornithorhynchus anatinus*).** Pairwise alignments were performed using LALIGN tool (https://embnet.vital-it.ch/software/LALIGN_form.html) in order to obtain the identity and similarity percentage between the two tested protein sequences.

| ***Gallus gallus*** | **AA Identity** | **AA Similarity** |
| --- | --- | --- |
| OCX-36 vs TENP | 19% | 53.8% |
| OCX-36 vs BPIFB6 | 24.3% | 60.2% |
| OCX-36 vs BPIL3 | 29.6% | 65.2% |
| OCX-36 vs BPIFB4 | 29.2% | 55.4% |
| OCX-36 vs BPIFB4-like platypus | 32.8% | 68.5% |
