# Supplementary material for: Evolution of the Avian Eggshell Biomineralization Protein Toolkit – New Insights From Multi-Omics
Source: Front Genet. 2021 May 11;12:672433. doi: 10.3389/fgene.2021.672433 (PMC8144736; doi:10.3389/fgene.2021.672433)
Supplement: Supplementary file 10 [file Table_4.DOCX]

**Supplementary Table 4:** **Identity and similarity between OC-17/OC-17-like/XCA proteins and REG4 in chicken (*Gallus gallus*) and Guinea fowl (*Numida meleagris*).** Pairwise alignments were performed using LALIGN tool (https://embnet.vital-it.ch/software/LALIGN_form.html) in order to obtain the identity and similarity percentage between the two tested protein sequences.

| ***Pairwise comparison*** | **AA Identity** | **AA Similarity** |
| --- | --- | --- |
| Chicken OC-17 vs REG4 | 29.2% | 58.4% |
| Guinea fowl OC-17-like vs DCA-1-like | 36.7% | 55.8% |
| Guinea fowl OC-17-like vs REG4 | 27.6% | 53% |
| Guinea fowl DCA-1-like vs REG4 | 31.3% | 59.5% |
| Chicken OC-17 vs Guinea fowl OC-17-like | 59.2% | 75.9% |
| Chicken OC-17 vs Guinea fowl DCA-1-like | 39.1% | 61.6% |
| Chicken REG4 vs Guinea fowl REG4 | 88.6% | 97.5% |
